# Emerging and divergent roles of pyrophosphorylated nucleotides in bacterial physiology and pathogenesis

**DOI:** 10.1371/journal.ppat.1009532

**Published:** 2021-05-13

**Authors:** N. Y Elizabeth Chau, Shehryar Ahmad, John C. Whitney, Brian K. Coombes

**Affiliations:** 1 Department of Biochemistry & Biomedical Sciences, McMaster University, Hamilton, Ontario, Canada; 2 Michael G. DeGroote Institute for Infectious Disease Research, McMaster University, Hamilton, Ontario, Canada; 3 David Braley Centre for Antibiotic Discovery, McMaster University, Hamilton, Ontario, Canada; Geisel School of Medicine at Dartmouth, UNITED STATES

## Abstract

Bacteria inhabit diverse environmental niches and consequently must modulate their metabolism to adapt to stress. The nucleotide second messengers guanosine tetraphosphate (ppGpp) and guanosine pentaphosphate (pppGpp) (collectively referred to as (p)ppGpp) are essential for survival during nutrient starvation. (p)ppGpp is synthesized by the RelA-SpoT homologue (RSH) protein family and coordinates the control of cellular metabolism through its combined effect on over 50 proteins. While the role of (p)ppGpp has largely been associated with nutrient limitation, recent studies have shown that (p)ppGpp and related nucleotides have a previously underappreciated effect on different aspects of bacterial physiology, such as maintaining cellular homeostasis and regulating bacterial interactions with a host, other bacteria, or phages. (p)ppGpp produced by pathogenic bacteria facilitates the evasion of host defenses such as reactive nitrogen intermediates, acidic pH, and the complement system. Additionally, (p)ppGpp and pyrophosphorylated derivatives of canonical adenosine nucleotides called (p)ppApp are emerging as effectors of bacterial toxin proteins. Here, we review the RSH protein family with a focus on its unconventional roles during host infection and bacterial competition.

## Introduction

Bacteria use nucleotide second messengers such as cyclic AMP, cyclic di-AMP, cyclic di-GMP, and (p)ppGpp to respond and adapt to changes in their surroundings. Under nutrient-limiting conditions, energetically costly synthesis pathways for DNA, rRNAs, and ribosomal proteins are rapidly inhibited, and cellular resources are redirected towards the synthesis of stress resistance factors, amino acids, and carbon metabolism [1,2]. These physiological changes are accomplished during the stringent response by guanosine tetraphosphate (ppGpp) and guanosine pentaphosphate (pppGpp); referred to herein as (p)ppGpp.

**Fig 1:**
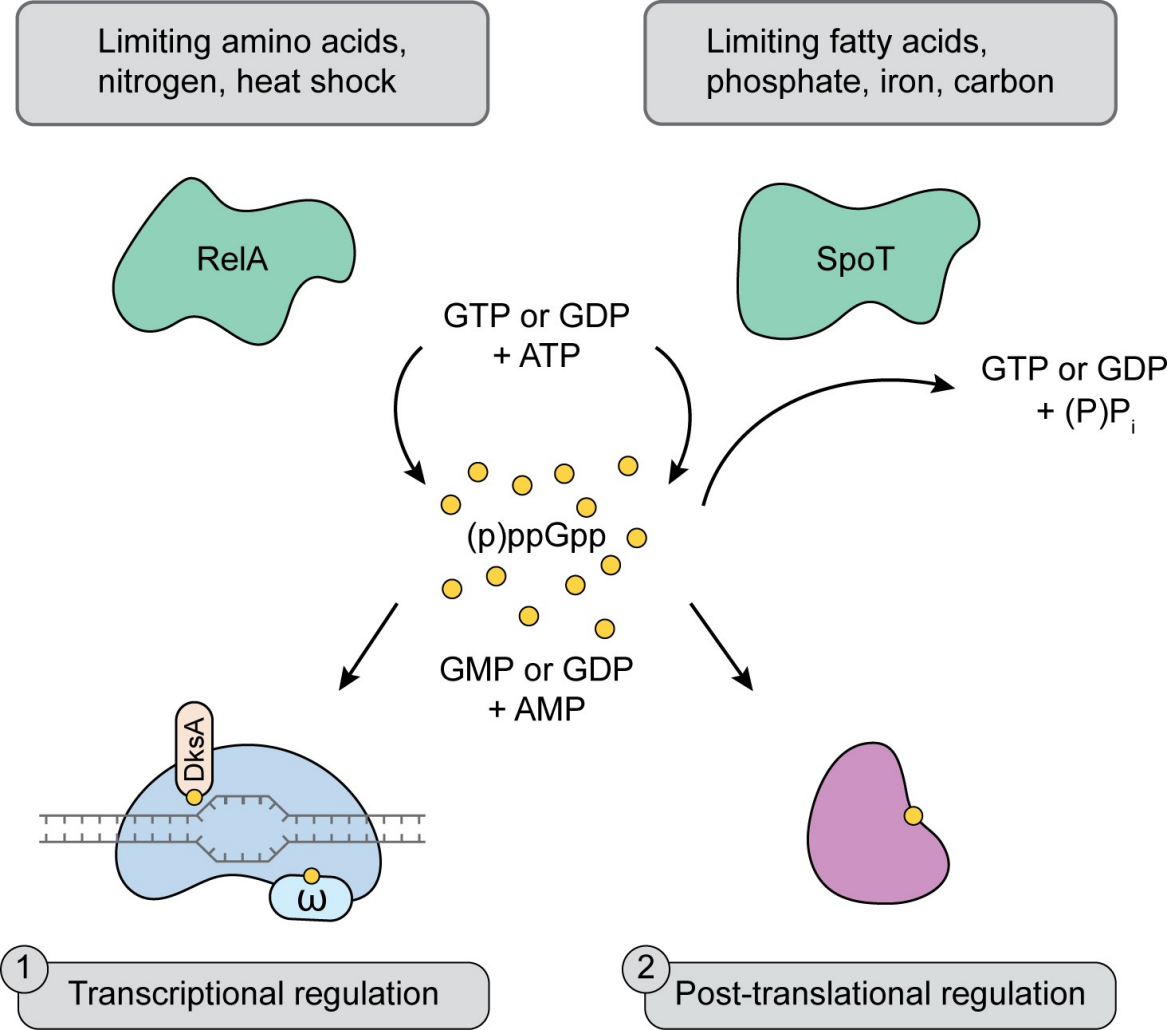
(p)ppGpp reprograms cell metabolism by transcriptional and post-translational regulatory mechanisms. In response to environmental stress, RelA and SpoT synthesize ppGpp and pppGpp by catalyzing the transfer of pyrophosphate from ATP to GDP or GTP, respectively, and generating AMP and GMP or GDP as byproducts. In contrast to RelA, SpoT is also able to hydrolyze (p)ppGpp to produce GTP or GDP and inorganic phosphate. ppGpp binds to RNA polymerase with DksA to modulate transcription, and to effector proteins to regulate their biochemical activity. AMP, adenosine monophosphate; DksA, DnaK suppressor A; GDP, guanosine diphosphate; GMP, guanosine monophosphate; GTP, guanosine triphosphate; ppGpp, guanosine tetraphosphate; pppGpp, guanosine pentaphosphate.

The cellular pool of (p)ppGpp is regulated by members of the RelA-SpoT homologue (RSH) protein family, which is comprised of small alarmone synthetases (SASs) and small alarmone hydrolases (SAHs), and multi-domain proteins containing both synthetase and hydrolase domains. Three multi-domain proteins called Rel, RelA, and SpoT are capable of synthesizing (p)ppGpp. (p)ppGpp is produced by the catalyzed transfer of a pyrophosphate moiety from ATP to the 3′ position of guanosine diphosphate (GDP) or guanosine triphosphate (GTP) [1–3]. In contrast to RelA, Rel and SpoT have a functional hydrolase domain that degrades (p)ppGpp back to pyrophosphate and either GDP or GTP [1–3]. Since the discovery of (p)ppGpp by Cashel and Gallant in 1969, extensive follow-up work has shown that (p)ppGpp coordinates adaptation to nutrient starvation through global transcriptomic reprogramming by directly interacting with RNA polymerase (RNAP) and several other downstream protein targets [4–8] (Fig 1).

In addition to coping with nutrient-related stresses, pathogenic bacteria employ (p)ppGpp signaling to regulate virulence gene expression. Evidence to support this includes the inability of bacteria devoid of (p)ppGpp ((p)ppGpp^0^) to swim, form biofilms, invade host cells, and resist innate immunity [2,9]. As a result, (p)ppGpp^0^ strains are significantly attenuated for fitness in animal models of infection [9]. Similarly, commensal microbes deficient in (p)ppGpp are also unable to survive and persist within the host environment [10]. Beyond the host-bacterial interface, bacteria and phages produce (p)ppGpp and pyrophosphorylated derivatives of adenosine nucleotides ((p)ppApp) to protect against phage superinfection and to compete with other bacteria, respectively [11,12]. Given the contributions of (p)ppGpp in regulating core cellular functions and virulence processes in bacteria, the stringent response has been identified as a unique target of interest for therapeutic inhibition. In this review, we highlight recent findings on the molecular regulation of RSH proteins, the role of (p)ppGpp during starvation, host infection, bacterial competition, and phage defense, and discuss the stringent response as an entry point to drug discovery in the age of antibiotic resistance.

### Distribution and regulation of RSH proteins

The genes encoding RSH proteins are broadly conserved in most species of bacteria with the exception of the phyla Planctomycetes, Verrucomicrobia, and Chlamydiae, and certain species of obligate intracellular endosymbionts [1,13]. Some eukaryotic organisms also contain RSH homologs. For example, *Drosophila melanogaster* expresses metazoan SpoT homologue 1 (MESH1) that functions as a ppGpp hydrolase and is likely involved in insect development and responses to nutrient starvation, whereas human MESH1 is a cytosolic NADPH phosphatase important for regulating ferroptosis [14,15]. Interestingly, although ppGpp has been detected in eukaryotic cell lines, a cognate synthetase has not been identified [16].

**Fig 2:**
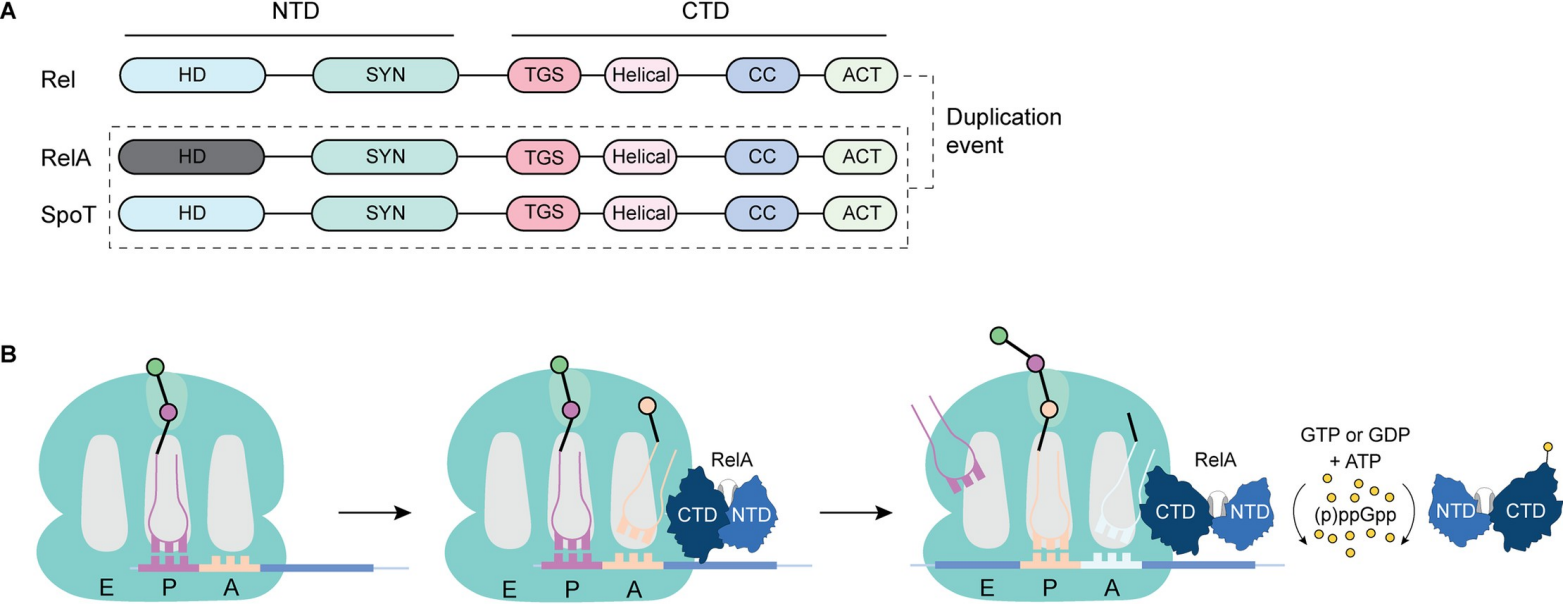
(p)ppGpp levels are regulated by RSH proteins. (**A**) Rel, RelA, and SpoT are long, multidomain RSH enzymes that synthesize (p)ppGpp. Rel and SpoT also have an active hydrolase domain that degrades (p)ppGpp. The domains of each RSH enzyme are depicted with the N-terminus comprised of the catalytic synthetase and hydrolase domains, and the C-terminus consisting of a TGS, a helical domain, a CC domain, and an ACT domain. (**B**) RelA monitors the translational status of the cell by directly associating with the ribosome. Accumulation of uncharged tRNAs in the ribosomal A-site triggers (p)ppGpp production. (p)ppGpp mediates positive feedback regulation of RelA, stimulating ribosome-independent (p)ppGpp synthesis. ACT, aspartokinase, chorismate mutase, and TyrA; A-site, acceptor site; CC, conserved cysteine; CTD, C-terminal domain; GDP, guanosine diphosphate; GTP, guanosine triphosphate; HD, hydrolysis domain; NTD, N-terminal domain; RSH, RelA-SpoT homologue; SYN, synthesis domain; TGS, ThrRS, GTPase, and SpoT; ThrRS, threonyl-tRNA synthetase.

The superfamily of enzymes that synthesize and degrade (p)ppGpp are named after RelA and SpoT, and the genes encoding both of these proteins are found in many species of β- and γ-Proteobacteria. RelA and SpoT likely originated from a gene duplication event from the ancestral, long multi-domain protein, Rel [1,13]. The N-terminus of Rel, RelA, and SpoT is comprised of a (p)ppGpp hydrolase domain (HD) and a (p)ppGpp synthetase domain (SYN), whereas the C-terminus consists of a threonyl-tRNA synthetase (ThrRS), GTPase, and SpoT domain (TGS), a helical domain, a conserved cysteine domain (CC), and an aspartokinase, chorismate mutase, and TyrA domain (ACT) [1,3] (Fig 2A). The functional role of the TGS, helical domain, CC, and ACT domains are not fully understood, but they have been suggested to regulate the enzymatic activities of the N-terminus via conformational changes, oligomerization, or interactions with other protein partners [17–27].

#### Bifunctional Rel

In species outside of β- and γ-Proteobacteria, (p)ppGpp is produced by the ancestral RSH protein, Rel, which maintains a close interaction with the ribosome to survey nutrient availability. (p)ppGpp synthesis occurs upon Rel detecting accumulated deacylated tRNAs in the ribosomal acceptor site (A-site). However, while this is a general mechanism, other cues can also stimulate Rel-mediated (p)ppGpp production. In *Caulobacter crescentus*, Rel requires carbon or nitrogen starvation in addition to amino acid deprivation to initiate the stringent response [28]. Biochemical and genetic analyses of the C-terminal domains (CTDs) of Rel enzymes have revealed molecular level insight into how the opposing synthetase and hydrolase functions of the N-terminal domain (NTD) are controlled [17]. In the absence of the ribosome, interaction of the TGS-helical domain of Rel with the NTD in *Bacillus subtilis* represses the synthetase domain while leaving the hydrolase domain active [18]. Recent structural studies on the NTD of Rel from *Thermus thermophilus* revealed an allosteric control mechanism mediated by (p)ppGpp and GTP. Binding of (p)ppGpp leads to a closed, inactive conformation, permitting only hydrolase activity, whereas GTP binding activates the synthetase domain [19]. In this way, the allosteric regulation of Rel allows for only one catalytic function to be active at a time, which prevents a futile cycle of (p)ppGpp synthesis and degradation. Other metabolites such as branched chain amino acids have also been shown to allosterically mediate the negative feedback mechanism of Rel activity in *Rhodobacter capsulatus* [20]. Valine and isoleucine are downstream products of the stringent response and their binding to the ACT domain of Rel increases its (p)ppGpp hydrolase activity. Interestingly, RelA from *E*. *coli* also has a strong binding affinity for valine, but it lacks hydrolytic function, suggesting that this may be an evolutionary relic following the duplication and divergence of Rel into RelA and SpoT [20].

#### Monofunctional RelA

Unlike Rel and SpoT, RelA has an inactive hydrolase domain and is the main enzyme involved in (p)ppGpp synthesis in *E*. *coli* and other β- and γ-Proteobacteria. During unstressed growth, it is proposed that the synthetase function of RelA is kept inactive through the formation of RelA homodimers that form via intermolecular disulfide bonds between adjacent CTDs [21–23]. However, this regulatory mechanism has been challenged by work showing that the expression of the CTD or full-length RelA does not compromise the ability of *E*. *coli* to respond to nutrient limitation nor does it result in a reduction in (p)ppGpp levels as would be expected if inactive dimers were formed [24]. Alternatively, it is possible that RelA remains as a monomer and is regulated by intramolecular interactions between the CTD and NTD [24]. The synthetase activity of RelA is activated upon exposure to various stresses including amino acid starvation, heat shock, and nitrogen stress [29–35]. Similar to Rel, RelA responds to the accumulation of deacylated tRNAs in the ribosomal A-site [29–32] (Fig 2B). One model suggests that (p)ppGpp synthesis occurs in a ribosome-independent manner and that RelA “hops” between ribosomes to monitor the translational status of the cell [36,37]. An extension of this model is that RelA retains “memory” of the starvation state and can continue to synthesize (p)ppGpp upon dissociating from the ribosome [38]. More recent studies suggest that RelA and deacylated tRNAs bind to the ribosome as a preformed complex, which, in turn, activates RelA-mediated (p)ppGpp synthesis. These findings are supported by a cryo-EM structure that shows the CTD of RelA interacting with the ribosome, whereas the N-terminal catalytic domain is flexible and protrudes away from the complex [39]. This is an area of ongoing investigation and more work examining the order and timing of RelA binding to the ribosome is still necessary to fully understand how it is activated. Additionally, RelA is regulated by (p)ppGpp through a positive feedback mechanism, which results in a transition to a ribosome-independent stimulation of enzyme function [40].

#### SpoT

SpoT functions primarily as a hydrolase for (p)ppGpp; however, it is also capable of mediating (p)ppGpp synthesis in response to signals distinct from those that activate RelA. Proteobacteria that contain RelA also have a hydrolytically active SpoT to prevent the accumulation of toxic levels of (p)ppGpp [41]. While the hydrolase activity of SpoT is essential for survival, mutations in regulatory domains that abrogate its synthetase function, particularly at the C-terminus, are well tolerated [42]. SpoT synthesizes (p)ppGpp in response to diverse stress signals including carbon, iron, phosphate, and fatty acid starvation [25,43,44]. The mechanisms by which SpoT senses different stress cues are not fully understood, but studies suggest that direct interactions with other cytosolic proteins play an important role in modulating its relative synthetase and hydrolase activities. Under nutrient-rich conditions, binding of the GTPase Obg to SpoT is thought to repress its (p)ppGpp-synthetic activity, whereas during fatty acid starvation, SpoT interacts with acyl carrier protein (ACP) and YtfK to activate (p)ppGpp synthesis [26,27]. During carbon source downshift, Rsd binds to the TGS domain of SpoT to stimulate its hydrolase activity, thus preventing toxic accumulation of (p)ppGpp [25].

### Molecular targets of (p)ppGpp signaling under nutrient stress

While the general physiological role of (p)ppGpp in promoting cell survival under stress has been well established in a number of bacterial species, characterizing the molecular targets (effectors) of (p)ppGpp and how its binding to these targets regulates their activities remains an active area of investigation. Two approaches have been used for the systematic identification of protein targets regulated by (p)ppGpp in *E*. *coli* [7,8]. The first method used an ordered overexpression library and measured the dissociation constants of each expressed protein with (p)ppGpp using a differential radial capillary action of ligand assay (DRaCALA) [8]. However, the use of DRaCALA for (p)ppGpp effectors failed to identify multiple proteins that have been validated in vitro, demonstrating that this technique may have a high false-negative rate [8]. In an alternative approach, photocrosslinkable ppGpp-peptide conjugates were used to affinity purify over 50 ppGpp targets from *E*. *coli*, the identities of which were determined by mass spectrometry [7]. Affinity capture provides a robust approach to identify (p)ppGpp effectors in organisms with a poorly characterized (p)ppGpp regulon and may also facilitate the discovery of new targets in organisms like *B*. *subtilis*, which has a well-characterized stringent response [45–47].

Most of the (p)ppGpp effectors characterized to date have been found in *E*. *coli* or *B*. *subtilis*. Despite differences in the specific functions of effector proteins, the effect of (p)ppGpp is similar in both species whereby upon starvation, the concentration of (p)ppGpp rapidly increases from micromolar to millimolar levels, leading to cellular reprogramming of pathways involved in DNA replication, transcription, translation, and nucleotide metabolism [7]. The majority of physiological and phenotypic changes that occur are due to transcriptional repression of energetically costly pathways such as rRNA and nucleotide synthesis, and activation of amino acid biosynthesis and uptake [48,49].

In *E*. *coli* and most other Proteobacteria, transcriptional changes are induced through direct binding of RNAP by (p)ppGpp, whereas in *B*. *subtilis* and other Gram-positive bacteria, changes in GTP homeostasis indirectly activate transcription factors [47,50–52]. In *E*. *coli*, (p)ppGpp binds to RNAP at 2 distinct sites with the first site being located at the interface of the RNAP β′ pincer and its ω domain while the second site is found at the binding interface between RNAP and the transcription initiation factor, DnaK suppressor A (DksA) [51,53,54] (PDB: 4KJR, 1TJL). The binding affinity of (p)ppGpp for site 1 is higher than site 2; however, binding at site 2 has a greater effect on RNAP activity, as it enhances the inhibitory effects of DksA on transcription. Thus, site 1 is likely important for ppGpp-binding in nonstarving, low (p)ppGpp states, whereas (p)ppGpp binding to site 2 takes place under starvation conditions and promotes global transcriptional changes in the cell. Transcriptional reprogramming in *E*. *coli* is also achieved in part by (p)ppGpp inhibiting RNAP from binding to the housekeeping sigma factor, σ^70^ [55]. As a result, RNAP is available to bind to the alternative sigma factors, σ^32^ and σ^38^, which are involved in activating genes required for adaptation to heat shock or nutrient limitation, respectively [55]. Moreover, consistent with the role of (p)ppGpp in modulating transcription by interacting with RNAP, a suppressor screen conducted on minimal medium with an *E*. *coli* strain unable to produce ppGpp (ppGpp^0^) selected for mutations in RNAP that rescued growth [56]. However, a similar screen using a ppGpp^0^ strain of *B*. *subtilis* selected for mutations that reduced GTP levels [57]. Indeed, many Gram-positive bacteria lack DksA and do not coordinate the stringent response directly through RNAP, but they instead rely on (p)ppGpp to regulate GTP levels in cells, which is accomplished through direct inhibition of purine metabolism [47,57]. At basal levels, (p)ppGpp prevents “death by GTP” by preventing an excessive rise in GTP levels [57,58]. At high (p)ppGpp concentrations, GTP levels are sharply reduced, and this primarily affects transcription through the activation of CodY, a transcriptional regulator in Gram-positive bacteria, for which GTP is a corepressor. This decrease in GTP levels reduces transcriptional activation of ribosomal genes that require GTP for initiation [57,59]. These data show that (p)ppGpp fine-tunes core cellular functions like transcription at incremental levels (see [60] for a recent review on the role of (p)ppGpp at basal levels).

Although the effects of (p)ppGpp on transcription are responsible for diverse phenotypes associated with the stringent response, (p)ppGpp also has many protein targets in the cell that belong to central metabolic pathways. Early work in the field showed that enzymes involved in purine metabolism are inhibited by (p)ppGpp in vitro and more recent studies provide a mechanistic basis for inhibition of this pathway, underscoring its role as a critical target of (p)ppGpp signaling [7,45–47,61,62]. In both *E*. *coli* and *B*. *subtilis*, (p)ppGpp inhibits enzymes in both the de novo and salvage pathways of purine nucleotide biosynthesis. Multiple phosphoribosyltransferases belonging to the purine salvage (HPRT, [45], PDB: 6D9S; XPRT, [46], PDB: 1Y0B) and de novo purine synthesis pathways (IMPDH and Gmk, [47], PDB: 4QRH) are targets of (p)ppGpp in *B*. *subtilis*. (p)ppGpp-mediated inhibition of these enzymes provides direct control of GTP levels, which, as mentioned previously, reprograms transcription in the presence of high (p)ppGpp levels. In *E*. *coli*, ppGpp inhibits purine metabolism enzymes like PurF ([7], PDB: 6CZF), Hpt/Gpt and Gsk ([62], PDB: 6VWP), and PpnN ([63], PDB: 6GFM). Recent work by Wang and colleagues (2020) showed that the inhibition of PurF, Hpt/Gpt, and Gsk leads to cellular conservation of the metabolite 5′ -phosphoribosyl-1′-diphosphate (pRpp), which is a precursor for purine and pyrimidine nucleotides, histidine, tryptophan, and NAD^+^. Consequently, when ppGpp levels increase in *E*. *coli*, the amount of purine nucleotides decreases. The resulting reduction in ADP specifically prevents the inhibition of the enzyme PrsA, which makes pRpp. This suggests that in addition to the inhibition of RNA synthesis through transcription, the inhibition of purine nucleotide metabolism allows for cells to conserve important metabolites [62].

Another mechanism mediated by (p)ppGpp that promotes bacterial survival during starvation is the inhibition of DNA replication elongation. It has been observed in both *E*. *coli* and *B*. *subtilis* that while the expression of the replication machinery is reduced upon (p)ppGpp production following starvation, the major inhibitory effect of these nucleotides on DNA replication is largely transcription independent [64–66]. Several groups have shown that this is due to (p)ppGpp-dependent inhibition of DNA primase, DnaG, which is a conserved (p)ppGpp target across several different species of bacteria (PDB: 4EDT) [66–68]. Inhibition of DNA primase likely hinders the synthesis of both the leading and lagging strands, which results in the rapid yet reversible arrest of DNA replication. This abrupt inhibition of replication may provide a way to preserve the genome during nutrient starvation, which, if left unregulated, may result in significant DNA damage [66].

Modulating protein translation rates is also a critical component of bacterial growth rate control. (p)ppGpp inhibits translation by directly binding to and inhibiting the enzymatic activity of initiation factor 2 (IF2). (p)ppGpp has also been shown to bind the elongation factors Tu and G [69–72] as well as several GTPases involved in ribosome biogenesis such as ObgE, RsgA, Rbg, and BipA [73] (ObgE, [74, 26], PDB: 1LNZ; RbgA, [75], PDB: 6G14; BipA, [76], PDB: 4ZCM). However, the physiological relevance of (p)ppGpp inhibition of these targets has not been explored in detail.

The characterization of (p)ppGpp targets thus far has provided important new insights into its extensive regulon. To date, there are limited examples of conserved motifs that would allow for the accurate prediction of (p)ppGpp protein targets using informatic approaches [46,62]. This lack of consensus (p)ppGpp-binding motif is consistent with the substantial differences in identified protein targets between different bacterial species [47]. The complexity of the (p)ppGpp signaling cascade warrants future efforts to both identify and characterize (p)ppGpp targets in other bacteria using approaches that combine biochemistry, structural biology, and genetics.

### Role of (p)ppGpp beyond nutrient limitation

The stringent response is commonly described as the rapid accumulation of (p)ppGpp during nutrient stress resulting in the inhibition of bacterial growth and conservation of key metabolic resources. Now, it is becoming increasingly appreciated that (p)ppGpp contributes to modulating additional cellular processes involved in host-bacterial, bacterial-bacterial, and bacterial-phage interactions.

#### (p)ppGpp at the host–pathogen interface

Beyond facilitating adaptation to nutrient limitation, the regulation of virulence factors by (p)ppGpp contributes to bacterial pathogenesis [2,9]. Here, we discuss the stringent response in the clinically relevant pathogens, *Salmonella enterica* serovar Typhimurium, *Mycobacterium tuberculosis*, *Enterococcus faecium*, and *Francisella tularensis*. We also encourage readers to see [2] for a recent review on the roles of (p)ppGpp in bacterial pathogenicity.

**Fig 3:**
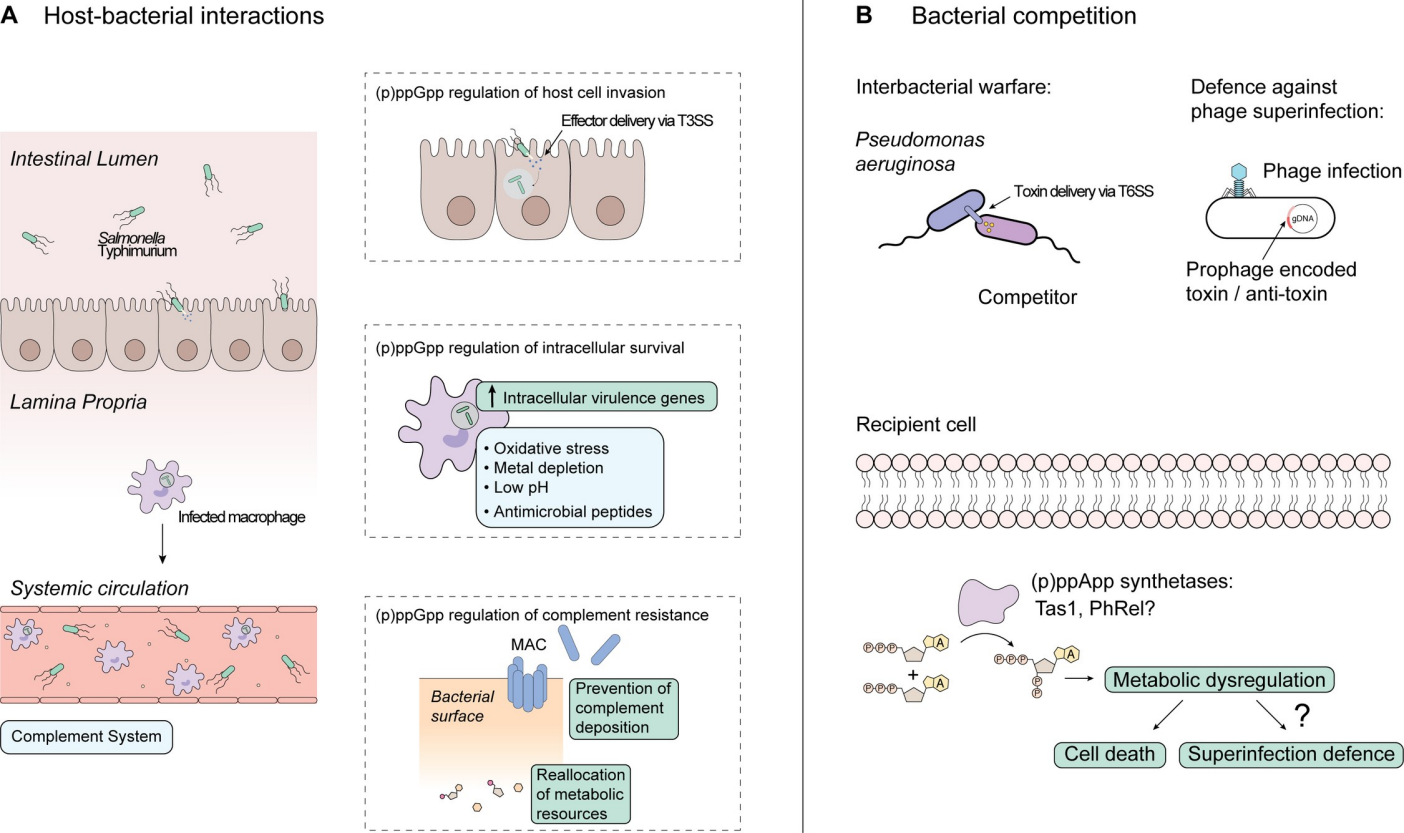
(p)ppGpp regulates gene expression during host-bacterial interactions, whereas (p)ppApp mediates bacterial competition. (**A**) (p)ppGpp contributes to activating genes required for host cell invasion and intracellular survival in *Salmonella* Typhimurium. Virulence gene regulation by (p)ppGpp also allows *S*. Typhimurium to evade different components of innate immunity including reactive nitrogen species, low pH, and the complement system. (**B**) (p)ppApp is used in interbacterial warfare and potentially in bacterial defense against phage superinfection. *Pseudomonas aeruginosa* delivers the toxin, Tas1, via a type VI secretion system into competitor bacteria. Tas1 produces (p)ppApp resulting in significant metabolic dysregulation and cell death. Prophage-encoded toxins such as PhRel may also produce (p)ppApp to confer protection against phage superinfection by reducing the metabolic potential of their host. MAC, membrane attack complex; T3SS, type III secretion system; T6SS, type VI secretion system; gDNA, genomic DNA.

In *S*. Typhimurium, it is well established that (p)ppGpp regulates genes required for the invasion of intestinal epithelial cells and intracellular survival (Fig 3A) [34,42,77–81]. Complementing these findings, recent work shows that the exposure of *S*. Typhimurium to nitric oxide (NO) induces amino acid auxotrophies that can be rescued by RelA-dependent activation of amino acid biosynthesis as well as expression of the flavohemoglobin protein, Hmp, which relieves NO stress [34]. (p)ppGpp also functions to subvert the complement system, a collection of proteins that target bacteria for phagocytic uptake or cell lysis [82]. In *Salmonella enterica* serovar Typhi, (p)ppGpp regulates extracellular capsule formation, which confers complement resistance and thus promotes serum survival [81]. However, *S*. Typhimurium lacks a capsule and (p)ppGpp instead regulates a nucleotide hydrolase called PpnN, and the biosynthesis of lipopolysaccharide O-antigen to confer complement resistance [8,63,83,84] (Fig 3A). The formation of biofilms is another mechanism that allows for bacterial evasion of complement and other environmental insults such as antibiotics [85,86]. Patients infected with *M*. *tuberculosis* require a long 6- to 9-month combination therapy in part because of nonreplicating persister cells that are commonly found in biofilms that can form in the lungs [87,88]. Moreover, the stringent response contributes to biofilm formation as strains of *M*. *tuberculosis* that are unable to synthesize (p)ppGpp showed a reduced ability to form biofilms [89]. Clinically relevant mutations affecting the production of (p)ppGpp can also arise during persistent infections such as in the case of a 6-week-old infant with leukemia infected with vancomycin-resistant *E*. *faecium* (VRE) [90]. Sequencing of the VRE isolates revealed a L152F missense mutation in the hydrolase domain of *relA* that resulted in constitutive activation of the stringent response. Notably, the mutant displayed significant tolerance to linezolid and daptomycin when grown in a biofilm compared to during planktonic growth [90]. These findings demonstrate that the stringent control of biofilms can provide pathogenic bacteria with a survival advantage during prolonged infection.

To persist within a host, some species of pathogenic bacteria have also developed the ability to reside in the intracellular environment of immune cells such as macrophages [91–93]. *M*. *tuberculosis* lacking the bifunctional Rel enzyme (Rel_Mtb_) or expressing a Rel_Mtb_ H80A variant, which retains synthetase activity but is unable to hydrolyze (p)ppGpp, is compromised for long-term survival during chronic infection in mice [89,94–96]. Mice infected with a Δ*rel*_Mtb_ mutant maintain nearly normal lung physiology with few granulomas containing aggregates of lymphocytes and foamy macrophages compared to mice infected with wild-type *M*. *tuberculosis* [96]. Consistent with these data, comparative transcriptomics of wild-type *M*. *tuberculosis* and a Δ*rel*_Mtb_ mutant showed significant down-regulation of genes for mammalian cell entry (*mce*) of macrophages and remodeling of the cell wall, which are required for its transition to an intracellular lifestyle [96]. A study done on *Francisella tularensis*, another intracellular pathogen, provides important mechanistic insights on how (p)ppGpp regulates virulence genes [93]. One mechanism of gene regulation involves the recruitment of σ^70^ to DNA by the heterodimeric complex, MglA-SspA, while another involves the binding of ppGpp to MglA-SspA to tether the transcription factor, PigR, to promoters and ultimately recruit RNAP [97,98]. Together, these mechanisms help to regulate the *Francisella* pathogenicity island (FPI), which encodes a type VI secretion system (T6SS) that is required for intramacrophage replication [99]. In *S*. Typhimurium, a transposon mutagenesis screen in J774 macrophages revealed that mutations at the 3′-end of *spoT*, which disrupt its C-terminal regulatory domain, were defective in adapting to acid stress [42]. Following phagocytic uptake, (p)ppGpp activates multiple two-component systems including PhoP-PhoQ and SsrA-SsrB, which are needed for *Salmonella* to resist intracellular host defenses [100,101]. Downstream of PhoP-PhoQ signaling, (p)ppGpp facilitates dimerization of SlyA, a transcription factor that contributes to coordinating resistance to low pH and cationic antimicrobial peptides [102]. However, this finding was recently challenged by conflicting data showing that the addition of (p)ppGpp did not induce SlyA dimerization nor did it affect its ability to bind DNA [103]. The activation of *ssrAB* by (p)ppGpp is thought to occur by relieving the negative repression on its promoter [80]. We and others have shown that SsrA-SsrB represses flagellar-based motility genes to facilitate evasion of inflammasome-mediated bacterial killing by the host [100,104,105]. Consistent with these data, *S*. Typhimurium develops a metabolically active, but slow- to non-growing state intracellularly and delivers effector proteins via its type III secretion system to reprogram macrophages into an anti-inflammatory state to evade immune killing [106,107]. In addition, it has also been suggested that (p)ppGpp is dispensable for inducing slow growth in *S*. Typhimurium and that environmental fluctuations such as low Mg^2+^ is sufficient [108]. Taken together, these studies highlight the complexity of virulence gene regulation and that it likely involves multiple factors including (p)ppGpp.

#### Distribution and functions of toxins that produce (p)ppGpp and (p)ppApp

In addition to long RSH enzymes, some species of bacteria encode SAS and SAH enzymes. By analyzing gene sequences in silico, many SASs were identified in two-gene operons characteristic of toxin-antitoxin (TA) systems, suggesting that (p)ppGpp production may be exploited by a toxin [11]. TA systems were originally discovered as plasmid maintenance modules consisting of a stable toxin and unstable antitoxin that results in the killing of plasmid-free cells post-segregation. However, there are also chromosomal TA systems, which act to modulate cell metabolism during environmental stress [109]. These findings are in line with the observation that the overproduction of (p)ppGpp inhibits bacterial growth. Indeed, 5 subfamilies of SAS-based toxins were identified and experimentally validated, and their toxicity was dependent on the (p)ppGpp synthetase active site, whereas SAHs can act as antitoxins [11]. A subset of these TA loci including gp29-gp30 is encoded among phage genes. gp29 is expressed by the mycobacterial prophage Phrann and was shown to be highly toxic when expressed in *Mycobacterium smegmatis*; however, the mechanism of its toxicity and its cognate anti-toxin, gp30, are unknown [110]. Notably, bacteria lysogenized by Phrann containing the gp29-gp30 TA system were able to prevent superinfection by lytic phages (Fig 3B). It is predicted that infection by lytic phages may induce dissociation of the gp29-gp30 complex and activate the (p)ppGpp-synthetase activity of gp29, leading to metabolic dormancy in the host bacterium and protection against superinfection [110].

Aside from (p)ppGpp-mediated regulation of intracellular processes, bacteria use diverse protein delivery systems to inhibit the growth of competitor bacteria [111]. For example, some species of bacteria encode T6SS to inject toxins and kill neighboring cells [112]. In *Pseudomonas aeruginosa*, a T6SS effector called Tas1 was shown to bear significant structural similarity to (p)ppGpp synthetases. However, biochemical analyses revealed that the toxin instead rapidly produces (p)ppApp and does not use GDP or GTP as substrates. This activity results in the depletion of ATP and causes global metabolic dysregulation in target cells [12] (Fig 3B). The toxicity of Tas1 can be alleviated by its cognate immunity protein, Tis1, which inhibits the toxin through direct binding. (p)ppApp-mediated toxicity can also be alleviated by the *P*. *aeruginosa* hydrolase, *Pa*SAH (formerly PA0431) [113]. *Pa*SAH may contribute to bacterial defense against Tas1-like effectors as the deletion of *Pa*SAH from a Tas1-sensitive *P*. *aeruginosa* strain renders it hypersusceptible to T6SS-delivered Tas1 [113]. In addition, and in contrast to (p)ppGpp, (p)ppApp is inefficiently degraded by *P*. *aeruginosa* SpoT, which results in (p)ppApp accumulation and likely contributes to its function as an energy depleting toxin [12]. Currently, the long RSH homologue from *Methylobacterium extorquens* and the small alarmone synthetase, SasA, from *B*. *subtilis* are the only other known enzymes that endogenously produce pyrophosphorylated derivatives of adenosine; however, these enzymes differ from Tas1 in that they also synthesize (p)ppGpp in response to stress [114,115]. (p)ppApp has also been reported in cell lysates of *E*. *coli*, but the identity of the enzyme responsible for its production is unknown [114]. While it remains understudied, (p)ppApp has been shown to bind to RNAP in vitro to mediate transcriptional changes; however, its binding site and effects on transcription differ from (p)ppGpp. Current evidence suggests that these nucleotides may have opposing functions in cells, for example, (p)ppGpp was shown to inhibit the canonical *rrnB* P1 promoter in vitro, whereas (p)ppApp stabilizes RNAP at this promoter and activates *rrnB* transcription [116]. However, both (p)ppGpp and (p)ppApp similarly regulate the metabolic enzyme PurF post-translationally, demonstrating that further studies are needed to compare the global effects of these nucleotide second messengers on bacterial physiology [7,8,12]. Although further investigation is needed to determine the full complement of cellular processes regulated by (p)ppApp, these recent studies expand the pool of nucleotide second messengers that are produced by bacteria.

### Targeting the stringent response for antimicrobial therapy

The rise in drug-resistant bacterial infections is causing a substantial burden on healthcare systems globally and requires innovative approaches for drug discovery. Many classes of antibiotics used in the clinic target essential cellular processes such as the cell wall or protein synthesis [117]. Recently, an alternative strategy that has emerged is the use of antivirulence compounds that disarm rather than kill the pathogen, which is thought to exert less selective pressure for resistance to develop [101,118]. Inhibition of the stringent response has been of interest for the development of antivirulence compounds owing to its involvement in regulating bacterial pathogenesis [1,2]. (p)ppGpp is also in part responsible for the induction of slow-growing subpopulations of bacteria that allow them to evade antimicrobial treatment regimes, adding to the ongoing health crisis [119,120]. Given that the role of (p)ppGpp in inducing persistence and tolerance has extensively been reviewed elsewhere [119–121], we will briefly focus on inhibitors of the stringent response here.

#### Finding novel inhibitors of the stringent response

One of the first inhibitors of the stringent response identified is a (p)ppGpp analog called Relacin, which prevents the formation of spores and biofilms by *B*. *subtilis* by inhibiting the (p)ppGpp synthetase activity of Rel. Although Relacin exhibits activity in vitro, it has a high half maximal inhibitory concentration (IC50) value of 200 μM, which would make it challenging to use in the clinic without potency improvements through medicinal chemistry [122]. As a result, this prompted the development of more effective and potent deoxyguanosine-based analogs of Relacin such as compound 2d [123]. Furthermore, a recent high-throughput screen was performed using the GlaxoSmithKline compound library to find inhibitors of recombinant Rel from *M*. *tuberculosis* [88]. This screening approach relied on the fluorescence detection of AMP released after Rel catalyzes the transfer of pyrophosphate from ATP to GTP or GDP. The screen led to the identification of compound X9, which was able to reduce fluorescence by 50% at 2 μM and directly kill nutrient-starved *M*. *tuberculosis*. By comparison, repression of fluorescence intensity in the Δ*rel* mutant required 16 μM of compound X9. Compound X9 also synergized with isoniazid, a conventional bactericidal drug, which demonstrates the potential for combinatorial treatment against tuberculosis [88]. While inhibiting RSH synthetase activity has shown promise, the full-length proteins are difficult to purify and are often unstable in vitro [40]. This makes it challenging to fully characterize their biomolecular interactions and enzymatic properties with small molecule inhibitors. The efficacy of small molecule inhibitors is also typically tested in whole cell assays against wild type and a mutant strain that is unable to synthesize (p)ppGpp. However, (p)ppGpp-deficient strains are prone to acquiring suppressor mutations that mimic the presence of (p)ppGpp, which may challenge the feasibility of cell-based approaches [60,124].

Another strategy that has been used to inhibit the stringent response is to target (p)ppGpp directly. The cationic synthetic peptide 1018 was reported to sequester (p)ppGpp and induces dispersal of biofilms formed by pathogenic bacteria such as *P*. *aeruginosa* [125]. However, it was later demonstrated that 8101, a control peptide with an inverted amino acid sequence to 1018 is equally potent and that 1018 does not display preferential activity against biofilms [126]. Instead, 1018 inhibited planktonic growth of *E*. *coli* in both (p)ppGpp inducing and noninducing minimal media. Other caveats to the specificity of 1018 include its potential use as an antiviral agent and its immunomodulatory properties affecting host chemokine responses and macrophage polarization [126,127].

## Conclusions

Our understanding of the stringent response has greatly expanded over the past 50 years. RSH proteins are broadly conserved across nearly all species of bacteria, demonstrating their pervasive role in bacterial physiology. Moreover, the molecular mechanisms underlying the cues that trigger (p)ppGpp production and govern the regulation of RSH enzymes are only beginning to be unveiled. (p)ppGpp was initially characterized in mediating adaptation to nutrient stress, but emerging research continues to highlight its role in regulating cellular homeostasis at basal levels as well as in response to diverse environmental stressors. Furthermore, its role beyond coordinating intracellular physiological processes is now being realized, with (p)ppGpp emerging as a major regulator of virulence gene expression in host-bacterial interactions. Additionally, the first physiological role for (p)ppGpp’s adenosine-containing counterpart, (p)ppApp, as a mediator of bacterial competition has been established. The widespread regulatory functions of (p)ppGpp have made it an attractive target for antimicrobial therapy, but efforts to identify inhibitors of the stringent response have seldom been successful. A more thorough investigation of (p)ppGpp signaling, and perhaps looking to natural products for potential inhibitors, may reveal new strategies to combat infections in the age of resistance.
